# Suicide ideation and/or attempt with substance use and associated factors among the youth in northwest Ethiopia, community-based

**DOI:** 10.1186/s12888-022-04157-x

**Published:** 2022-07-28

**Authors:** Mamaru Melkam, Demeke Demilew, Tilahun Kassew, Bruik Fanta, Sewbesew Yitayih, Kassahun Alemu, Yasin Muhammed, Berhanie Getnet, Eden Abetu, Gebrekidan Ewnetu Tarekegn, Mohammed Oumer, Goshu Nenko

**Affiliations:** 1 Department of Psychiatry, College of Medicine and Health Science, University of Gondar, Gondar, Ethiopia; 2 Department of Biostatistics & Epidemiology, College of Medicine and Health Science, University of Gondar, Gondar, Ethiopia; 3 Department of Psychology, College of Social Science and Humanities, University of Gondar, Gondar, Ethiopia; 4 Department of Clinical Pharmacy, College of Medicine and Health Science, University of Gondar, Gondar, Ethiopia; 5 Department of Human Anatomy, College of Medicine and Health Science, University of Gondar, Gondar, Ethiopia

**Keywords:** Substance use, Suicidal ideation and attempt, Youth, Ethiopia

## Abstract

**Background:**

Substance use is referring to the use of psychoactive substances like chat, cigarettes, alcohol, and others. The use of substances particularly (alcohol, chat, and cigarette) is a major mental health burden in developing countries including Ethiopia among youth. Suicide ideation and an attempt are thinking or trying to kill oneself that facilitates the act of a person intentionally causing his or her death. Suicide is one of the most serious mental health problems and has a great social impact in the world as it is currently the third leading cause of death for youth. Youth is defined as the period of life between childhood and maturity with an age interval of (15–25).

**Method:**

A cross-sectional study design was used to assess the prevalence of suicidal ideation and attempts with substance use among youth in northwest Ethiopia. Multi-stage sampling techniques of stratified with simple random sample ware used. In the first stage, substance users are selected then as the second stage among substance users the burden of suicide behavior is assessed. ASIST, DASS-21, and other tools were used to assess suicidal behavior with substance use and associated factors. Data were edited, purified, and entered into Epi-data version 4.6 before being exported to the statistical package for social sciences version 20 for analysis of bi-variables to see the associations’ *p*-value < 0.2 and multi-variables to identify the associated variables with a *p*-value of < 0.05 AOR and CI also done.

**Results:**

From a total of 372 substance user participants over all prevalence of suicidal ideation and attempt among youth was 54(14.5%) with 95% CI of (11.0,18.0) and 37(9.9%) with 95% CI (7.0, 13.0) respectively**.** Being female [AOR =2.36;95% CI:(1.19, 4.68)], poor social support [AOR =3.03; 95% CI: (1.11, 8.25)], and anxiety [AOR = 3.82: 95% CI; (1.96, 7.46)].

**Conclusion and recommendations:**

The prevalence of suicidal ideation and attempt among substance users was 14.5 and 9.9% respectively therefore, immediate interventional actions needed to be administered to decrease the burden of suicide by reducing substance use and other associated factors.

## Introduction

Substance abuse is a major global health problem related to multiple adverse mental health and social outcomes for the younger generation [1]. In most parts of the world, the use of the substance is a phenomenon that can result in displays in terms of both morbidity and death [2–4]. It has been established that substance use including cigarettes, alcohol, and other substances is a worldwide threat that affects young people [5]. Worldwide use of substances is estimated that alcohol causes 1.8 million or 3.2% of death and using tobacco is estimated to kill greater than 5 million people each year [6, 7]. The consumption of substances has different adverse effects including violence and suicide, particularly for youth [8]. Recently studies indicate the relationship between substance use and suicide needs to be solved to reduce its burden [9–11].

Suicidal ideation is defined as thoughts of self-killing or serving as the agent of one’s death intentionally the seriousness depends on the degree of intent and plan for the suicide attempt and completed suicide including youth [12]. There is a strong association between substance use and Suicidal among youth which brings ill-adjusted life [8]. Even though suicide has a significant public health impact can be reduced by minimizing the underline cause or precipitating factors like substance use. Knowing the linkage between substance use and suicide established to understand and apply the mechanism of designing a better management principle and guideline for appropriate interventions [11].

Suicide is the second leading cause of death for those between the ages of 15 and 29 worldwide, accounting for 1.4% of all deaths [13]. The magnitude of suicide has increased by 16.6% from time to time in the last decades [14]. According to the Mexican National Comorbidity Survey, the burden of suicidal behavior, including thought, planning, and attempt, is becoming more and more severe among people [15]. In Canada, suicidal behavior becomes the second leading cause of death unexpectedly before the natural pose 11.3 per 100,000 fatalities, however, it is the third leading cause of death in young people [16, 17]. Suicide may not always end with death which means there is non-fatal suicidal behavior after attempting it more commonly from 25 to 50 times [18]. Suicidal attempt from youth is a significant factors to accomplish [19]. The burden of suicidal attempts in the sub-Saharan African countries among youth was 28 .3% [20]. Even if the amount of substance particularly alcohol consumed at the time of a suicidal attempt is not exactly recorded it is one of the major triggering factors for youth.

Few reports demonstrate the connection between other substances and suicide but the consumption of alcohol and suicidal behaviors was well studied relatively [11]. Some substances might be used for direct suicide behavers to end life by taking an overdose, especially females attempt by posing with similar drug and there is limited suicidal management guideline for adolescents [21, 22].

Studies that try to estimate the prevalence of suicidal ideation and attempts among children and youth in sub-Saharan countries [23]. In Ethiopia’s meta-analysis and systematic review, the prevalence of suicidal ideation and attempted suicide ranged from 1 to 55 and 0.6% to 14% respectively. Another cross-sectional study in Ethiopia among students showed the prevalence of suicide ideation and attempts among study participants was found to be 14 to 58.3% and 4.4, to 7.4%, respectively [24–26].

Suicidal ideation and/or attempts were statistically significantly correlated with living alone, depression, depressed mood, family history of mental illnesses, prior psychiatric disorders, gender, poor social support, feeling neglected by parents, the end of a committed romantic relationship, and poor physical health [24, 27]. Living alone, depressed mood, family history of mental illnesses, previous psychiatric disorders, gender, poor social support, feeling neglected by parents, the breaking of a steady love relationship, and poor physical health were statistically significantly associated with suicidal ideations and/or attempts [24–28]. Little is known about suicide ideation and behavior among youth especially since there is no study conducted at the community level. The present studies show the burden of suicide only, the relationship between substance use and suicide is not yet found. Therefore, the major objectives of this study were to determine the burden of suicidal ideation and/or attempts with substance use among youth at the community level in Ethiopia.

## Methods and materials

### Study area

Suicidal ideations and/or attempt with substance use were conducted in Central Gondar Zone Northwest Ethiopia. Central Gondar Zone is located in the Northwest of Ethiopia in Amhara regional which area covers 21,791.83 KM^2^. In central Gondar is a newly formed zone from the previous North Gondar zone. In the central Gondar zone, there are 16 districts (15 rural districts and Gondar special district) and 442 kebeles. According to the 2012 E. C population statistics the central Gondar zone population was estimated at 2,642,138. Of this, 575,656 young people (15 to 24 years old) made up 21.79% of the overall population. Males and females make up 286,385 and 289,271 of the youth, respectively.

### Source and study population

#### Sources population

All youth (15–24 years old) who were living in Central Gondar Zone.

#### Study population

All youth in the Central Gondar zone who were living in the selected Kebeles.

### Inclusion and exclusion criteria

#### Inclusion criteria

All youth aged (15–25 years old) were included in the study.

#### Exclusion criteria

Youth who were unable to communicate due to severe mental/ physical illness were excluded.

### Sample size and sampling technique

The sample size was determined by two stages, the first stage was calculated to determine the prevalence of substance use and the second stage was to determine the prevalence of substance-induced suicidal ideation and attempts among substance users as described below.

**In the first stage,** the required sample for this study was determined by using both single population proportion formulas and two population proportion formulas. A high value was taken from the single and two population proportion formula to get the maximum sample size. Epi-Info software was used to calculate and the following formula was also used to calculate manually.

$$\frac{\mathrm{n}={\left({\mathrm{Z}}_{\upalpha /2}\right)}^2\mathrm{p}\left(1-\mathrm{p}\right)}{{\mathrm{d}}^2}$$

Where:p = estimated prevalence was taken from the previous study conducted in Woldiya town preparatory school among student on the current substance use (34.6%) [29].d = Margin of error (d) =3% = 0.04.Z𝘢/2 = Z value at (α = 0.05) = 1.96 corresponding to 95% confidence level.

$$\frac{(1.96)^2\mathrm X\;0.346\left(1-0.346\right)}{(0.04)^2}=\underline{598}$$

Since we have used the multistage sampling technique design effect of two is multiplied by the calculated sample which gives 1196. Then adding 10% (1196 × 0.1 = 119.6 ≈ 120) as a non-response rate the total sample size for prevalence is **1196 + 120 = 1316.** Therefore, the minimum required to sample for this study is **1316** (Table 1).

**Table 1:** Calculating sample size by considering associated factors for the estimation of minimum required sample size for the study

	Variable	Exposed	Unexposed	OR	Sample size
1.	Age	> 19 years (8.5%	15–19 years: 26%	1.82	573
2.	Friends use (yes)	Yes: 29%	No: 5.3%	7.42	191

**Fig. 1:**
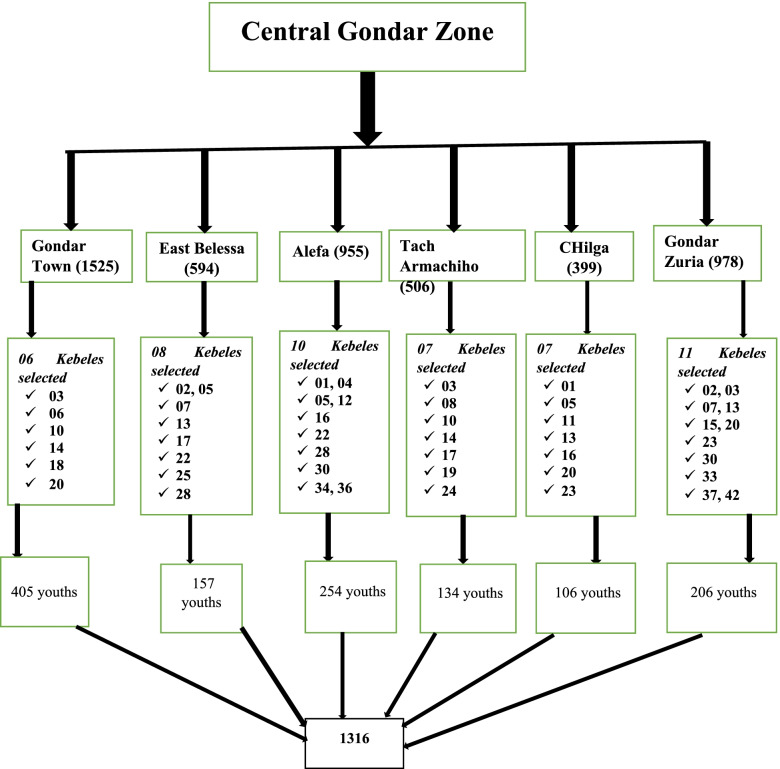
Schematic presentation of sample for the selecting the study participants in Gondar zone

Our study region is large, making a single stage challenging, hence the multistage sampling technique was adopted. To provide everyone an equal opportunity of being chosen, the zone was a geographic cluster based on each district’s woredas and then down to Kebele. The number of participants at each randomly selected kebele were allocated proportionally based on the size of the youth to be obtained from the central Gondar zone. Finally, from the randomly selected Kebeles, participants were recruited from Ketenas (Gott) inside Kebele until the proportional sample was filled with a cluster sampling technique which was surveyed randomly in each stage based on the rule of thumb (Fig. 1).

**In the second stage,** the participant were one or more substance users in the last 3 months from the whole sampled youth. From the first stage calculated sample (1316), the substance users considered whether they have suicidal ideation or attempt with substance use. Therefore, from the above (1316) recreated samples 370 were current substance users that are considered for the further analysis of substance-induced anxiety disorders.

### Operational definitions

**Suicidal ideation:** is the respondents, answer the question, have you seriously thought about killing self in the last one month? If yes, the respondent is considered as experiencing suicidal ideation.

**Suicidal attempt**: is defined as, if the respondents, answer the question have you attempted suicide in the last one month? If yes, the respondent is regarded as experiencing a suicidal attempt [30].

**Youth:** denote the late adolescent and young adult (15–25 years old) that developmental dramatic change takes place like rapid physical growth, cognitive and moral development as well as emotional development and change. This age category needs properly managed unless youth may prone to risk-taking behaviors, including substance use [31].

**Ever substance use:** based on ASSIST individuals who use at least one specific substance once in their life like alcohol, tobacco, and cigarette.

**Current substance use:** means based on ASSIST individuals who use at least one of the specified common substances in the last three months [32].

**Anxiety:** is defined based on the DASS-21 anxiety subscale, individuals who scored 15 or more were considered to have anxiety.

**Stress:** is defined based on the DASS-21 stress subscale, individuals who scored greater than or equal to 26 are considered as having stress [33].

**Perceived social support:** as MSPSS-12 items, any means scale score ranging from 1 to 2.9 could be considered low support; a score of 3 to 5 could be considered moderate support; a score from 5.1 to 7 could be considered high support [34].

### Study variables

#### Dependent variable

Suicidal ideation and/or attempt with substance users.

#### Independent variables

Personal characteristics: Gender, age, religion, marital status, educational status, occupation, residency, and current living arrangement. Family-related variables: family size, father’s/mother’s level of education, occupational status of father/ mother, biological parents alive, having a family member using a substance, the recent loss of loved ones, and having a friend using a substance. Psycho-social variables: Perceived social support, level of psychological stress. Substance use: such as alcohol and chat, and cigarettes.

### Data collection procedure and tool

The **first part** of the data was a socio-demographic questionnaire that include basic demographic and family-related characteristics of participants.

The **second part** of the data was the Alcohol, Smoking, and Substance Involvement Screening Test (ASSIST) questionnaire that was used to assess risky substance use. The ASSIST consists of eight questions covering tobacco, alcohol, cannabis, cocaine, amphetamine-type stimulants, inhalants, sedatives, hallucinogens, opioids, and other drugs. The WHO recommended ASSIST cut-off scores for conventional risk levels (low, moderate, high) are as follows: for alcoholic beverages: low risk (0–10), moderate [11–26], and high risk (27^+^).

Substances other than alcohol are categorized based on the following scores low (0–30, moderate [4–26], and high risk (27^+^).

The **third part** Depression, Anxiety and Stress Scale-21 Items (DASS-21) were used to assess the presence of depression, anxiety, and stress. Each of the DASS-21 scales contains 7 items, divided into subscales with similar content. The responses for each statement scored on a Likert scale from 0 to 3 which indicates how much the statement applied to you over the last week. DASS-21 needed to be multiplied by 2 to calculate the final score.

The recommended cut-off scores for conventional severity labels (normal, moderate, severe) are as follows:

✓ Anxiety: 0–7 normal; 8–9 mild; 10–14 moderate and 15 and above severe.

✓ Stress: 0–14 normal, 15–18 mild, 18–25 moderate, and score greater than 26 severe.

✓ Depression: 0–9 rated as normal; 10–13 mild, 14–20 moderate; 21 and above severe.

The **fourth part** of the questionnaire was the Multidimensional Scale of Perceived Social Support (MSPSS) contains a 12-item scale designed to measure perceived social support from three sources: Family, Friends, and a Significant Other. The scale is comprised of a total of 12 items, with 4 items for each subscale.

Significant Other Subscale: Sum across items 1, 2, 5, & 10, and then divide by 4. Family Subscale: Sum across items 3, 4, 8, & 11, and then divide by 4. Friends Subscale: Sum across items 6, 7, 9, & 12, and then divide by 4. Total Scale: Sum across all 12 items, then divide by 12. In this approach any mean scale score ranging from 1 to 2.9 could be considered low support; a score of 3 to 5 could be considered moderate support; a score from 5.1 to 7 could be considered high support.

### Data quality assurance

The questionnaire was translated by bilingual experts after first being prepared in the English language, then back-translated Amharic language. 15 data collectors (BSc in Psychiatry) and 5 supervisors (mental health professionals) were selected, and training was provided for a 2 half days duration about data collection tools, collection techniques, and ethical issues during the selection and collection of the data. The supervisors have assessed the consistency and completeness of data on daily basis. A pre-test of all structured questionnaires was checked on 5% of young people before the main data collection. A pre-test was conducted in the North Gondar which is out of the study area among youth.

### Data analysis procedure

The data was entered into the appropriate statistical software program (Epi-Data 4.6 version) and then exported into SPSS version 20 for further analysis. Descriptive statistics were used to measure the summarize the statical data distribution. Bi-variable logistic regression was conducted to check the associated factors with suicidal ideation and/or attempt with a *p*-value of < 0.2 to be reanalysis in multi-variable regression. Multi-level binary logistic regression analysis was performed to assess the significant associated factors of suicide ideation and/or attempt. Adjusted odds ratio with 95% confidence interval was used to declare statistically significant variables based on *p* < 0.05 in the multivariable logistic regression model. The basic assumptions and model fitness of the statistical analysis model used in this study was 75.30 on Hosmer and Lemeshow test. The absence of collinearity among independent variables was determined by VIF, which means there is no collinearity since it is greater than ten. Therefore, the outcome variable was distributed normally with the determinant factors.

## Results

### Social-demographic characteristics of participants

Of a total of 1316 participants, 372 were substance users at least one of them for the last three months to be included for the analysis of suicidal ideation and/ or attempt. Of the respondents 274 (73.7%) were male and the mean age of participants was 20.51 with a standard deviation of **±**2.605. Most of the study participants were 316(84.9%) Orthodox and 48(12.9) Muslim religious followers. Of the participant, 184(49.5%) were high school students and a total of 217(58.3%) were students including high school students but 55(14.8%) were a merchant. More than half of the respondents 222(59.7%) were from urban residences and 266(71.5%) were living alone. Among the study participants,143(38.4%) had a family who use alcohol and 181(48.7%) had a friend who use substances (Table 2).

**Table 2:** Sociodemographic characteristic of study participants among youth in Northwest Ethiopia

Variables	Category	Frequency	Percentage
Sex	Male	274	73.7%
	Female	98	26.3%
Age new	15–19	149	40.1%
	20–24	210	56.5%
	25	13	3.5%
Marital	Married	310	83.3%
	Single	55	14.8%
	Others^a^	7	1.9%
Religion	Orthodox	316	84.9%
	Muslim	48	12.9%
	Others^b^	8	2.1%
Education	Unable to write/ read	31	8.3%
	1st school	79	21.2%
	2nd school	184	49.5%
	Diploma/ degree	78	21.0%
Job	Government employed	30	8.1%
	Merchant	55	14.8%
	Farmer	19	5.1%
	Students	217	58.3%
	Others^c^	41	13.8%
Residence	Urban	222	59.7%
	Rural	150	40.3%
Living with	With family	95	25.5%
	Alone	266	71.5%
	Other^d^	11	3.0%
Father education	Uneducated	201	54.2%
	1St school	125	33.7%
	2nd school	23	6.2%
	Diploma/ above	22	5.9%
Mother education	Uneducated	265	71.4%
	1St school	72	19.4%
	2nd school	16	4.3%
	Diploma/ above	18	4.9%
Alcohol use by family	Yes	229	61.6%
	No	143	38.4%
Loss	Yes	109	29.3%
	No	263	70.7%
Friend use of substances	Yes	181	48.7%
	No	191	51.3%

Other^a^ = divorced, widowed

Others^b^ = protestant, catholic

Others^c^ = labor workers, housewife

Others^d^ = live with other relatives

### Psychological factors

Among the participants 47(12.6%) had poor social support, 195(52.4%) had moderate social support and 130(34.9%) had high social support. Of the respondents, 10(2.7) had stress (Table 3).

**Table 3:** Psychosocial factors of study participants among youth in Northwest Ethiopia

Variables	Categories	Frequency	Percentage
Social support	Poor	53	14.2%
	Moderate	192	51.6%
	High	127	34.1%
Anxiety	No	262	70.4%
	Yes	110	29.6%
Depression	No	297	79.8%
	Yes	75	20.2%
Stress	No	362	97.3%
	Yes	10	2.7%

### Prevalence of suicidal ideation and attempt

**Fig. 2:**
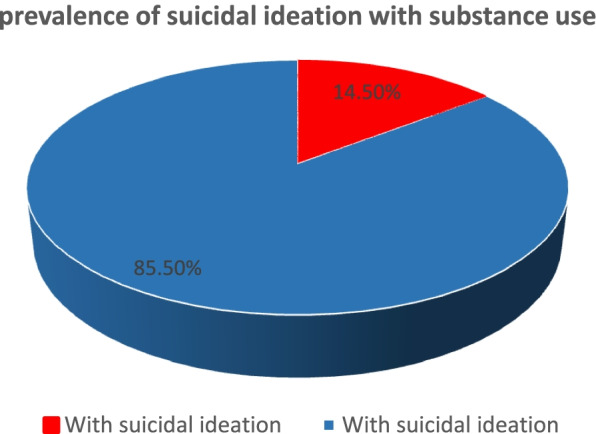
Prevalence of suicidal ideations with substance uses among youths in Gondar zone

**Fig. 3:**
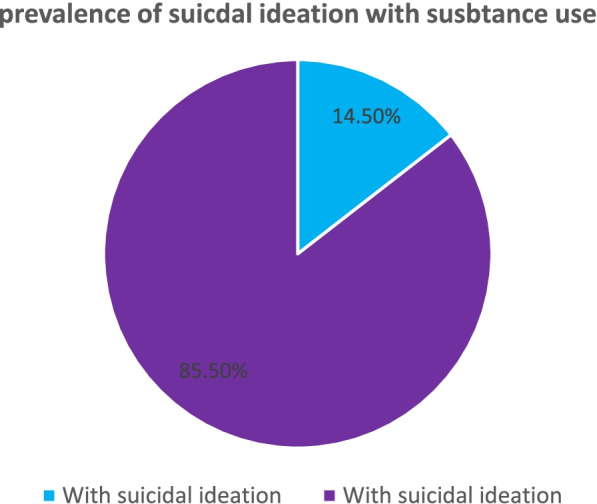
Prevalence of suicidal attempt with substance use among youths in Gondar zone

The prevalence of suicide ideation and attempt among substance users’ youth were 54(14.5%) with 95% CI of (11.0,18.0) and 37(9.9%) with 95% CI (7.0, 13.0) respectively (Figs. 2 and 3).

### Associated factors of suicidal ideations and attempt

From bi-variables logistic regression analysis being female, age, alcohol use family, friend’s use of the substance, poor social support, being stressed, anxiety and depression were associated with suicidal ideation with a *p*-value of less than 0.2. From bi-variables multi-variables logistic regression being female, poor social support, and presence of anxiety were associated with suicidal ideation with a *p*-value of less than 0.05.

From the associated factors being females was 2.36 time more likely to develop suicidal ideation as compared with male participants [AOR =2.36: 95% CI:(1.19, 4.68)].

The probability of developing suicidal ideation was 3.03 times more with poor social support as compared to other participants [AOR =3.03; 95% CI: (1.11, 8.25)].

The probability of developing suicidal ideations among anxious youth was 3.82 times more likely as compared to the participant without anxiety [AOR = 3.82: 95% CI; (1.96, 7.46)] (Table 4).

**Table 4:** Regression table of suicidal ideations with substance users among youth in Northwest Ethiopia

Variables	Category	Suicidal ideation of sub. users		COR with 95% CI	AOR with 95% CI
		Yes	No		
Sex	Male	34	240	1	1
	Female	20	78	1.81(0.89, 3.32)	2.36(1.19, 4.58) *
Age	15–19	14	135	1	1
	20–24	34	176	0.12(0.36, 0.41)	1.51(0.75, 3.04)
	25	6	7	0.23(0.07, 0.71)	0.88(0.07, 11.24)
Alcohol use of family	Yes	38	191	1.58(0.84, 2.95)	0.60(0.29, 1.25)
	No	24	167	1	1
Friends sub. Use	Yes	30	151	0.72(0.40, 1.29)	1.17(0.59, 2.31)
	No		25	1	1
Social support	Poor	13	40	4.26(1.69, 10.72)	3.02(1.11, 8.25) *
	Moderate	32	160	2.62(1.20, 5.70)	1.76(0.82, 3.80)
	High	9	127	1	1
Stress	Yes	4	6	4.16(1.13, 15.26)	3.19(0.65, 15.45)
	No	50	312	1	1
Anxiety	Yes	30	80	3.72(2.05, 6.73)	3.82(1.96, 7.46) *
	No	24	218	1	1
Depression	Yes	11	64	1.01(0.49, 2.08)	0.43(0.17, 1.05)
	No	43	254	1	1

* significantly associated

Form bi-variables logistic regression analysis sex, marital status, father’s education, mother’s education, alcohol users’ family, loss of loved one, anxiety, and poor social support were associated with suicidal attempts with a *p*-value of less than 0.2. From bi-variables multi-variables logistic regression analysis being female and poor social support wear associated with suicidal attempts with a *p*-value of less than 0.05.

Of significantly associated factors being female was 2.56 times more at risk to develop suicidal attempts than male youth participants [AOR = 2.56; 95% CI: (1.14, 5.73)].

The probability of development of suicidal attempts among youth participants with poor social support was 6.46 times more likely as compared to other participants [AOR = 6.46; 95% CI: (2.35, 17.8)] (Table 5).

**Table 5:** Regression table of suicidal attempts with substance use among youth in Northwest Ethiopia

Variables	Category	Suicidal attempt of sub. Users		COR with 95% CI	AOR with 95% CI
		Yes	No		
Sex	Male	23	251	1	1
	Female	14	84	1.82(0.89, 3.69)	2.36(1.19, 4.58) ^a^
Marital status	Married	26	278	1	1
	Single	5	51	0.54(0.00, 0.60)	0.83(0.26, 2.59)
	others	6	6	0.04(0.00, 0.53)	2.17(0.14, 32.8)
Alcohol use of family	Yes	17	212	0.49(0.25, 0.97)	1.56(0.71, 3.42)
	No	20	123	1	1
Father education	Uneducated	18	183	0.33(0.11, 1.01)	0.22(0.04, 1.14)
	1St school	11	114	0.32(0.10, 1.06)	2.17(0.14, 3.80)
	2nd school	5	20	0.51(0.10, 2.45)	3.99(0.53, 4.25)
	Diploma/ above	3	17	1	1
Mother education	Uneducated	20	241	0.35(0.10, 1.14)	1.80(0.28,11.40)
	1St school	8	64	0.44(0.12, 1.66)	1.72(0.26, 11.42)
	2nd school	5	15	0.23(0.02, 2.35)	0.43(0.03, 5.71)
	Diploma/ above	4	14	1	1
Social support	Poor	16	32	4.63(2.55, 16.22)	6.46(2.34, 17.82)
	Moderate	13	179	1.08(0.43, 2.68)	0.99(0.37, 2.60)
	High	8	119	1	1
Anxiety	Yes	13	97	1.32(0.65, 2.71)	1.41(1.62, 3.18)^a^
	No	24	238	1	1
Loss of loved one	Yes	12	97	0.84(0.41, 1.75)	1.26(0.55, 2.92)
	No	25	238	1	1

^a^Significantly associated

## Discussions

The prevalence of suicidal ideations and attempt with substance users among youth were 54(14.5%) with 95% CI of (11.0,18.0) and 37(9.9%) with 95% CI (7.0, 13.0) respectively. The suicidal attempts occurred after the existence of suicidal ideation which indicates the severity of the behaviors.

The prevalence of both suicidal ideation and attempt were in line with other studies conducted in Ethiopia 14 and 7.4% respectively [24], France 15.2 and 6.3% [35], and Bangladesh 12.8% [36]. In another way, the prevalence of suicidal ideation and attempt was higher than in other studies conducted in China 2.7% [37], pooled China study 2.8% [38]. The discrepancy might be explained by the other studies conducted without considering substance users but our study is assessed among substance users increase the risk of suicidal behaviors. The finding of this study also showed the prevalence of suicidal ideation and attempt was lower than in other studies conducted in Jamerica at 38 and 25% [39]. The possible reason for the discrepancy might be due to the effect of different sitting and sociodemographic factors on the participant in developed countries as compared to Ethiopian youth [38]. The other reason could be the evidence of the presence of high social functioning and religiosity in the community.

There are several factors associated with both suicidal ideation and attempt from substance users among youth. Being female was one of the factors associated with both suicidal ideation and attempt. This finding is consistent with other studies conducted in [24, 40–42].

The probable evidence of the association might be because females are more depressed and anxious as compared to males [40, 41]. The use of substances-induces suicide due to directly causing suicidal thoughts, especially alcohol, and indirectly by increasing the other factors like stress and anxiety finally leading s to suicidal behaviors [40]. Females are less tolerant of emotional stress and challenges due to the effect of a culture that prohibited them to reflect on their intention could be the other evidence that leads to suicidal behavior [42]. The vulnerability of psychosocial distress includes depression due to the hormonal difference, stress, and behavioral model of learned helplessness [24, 43].

Participants who had poor social support were the other associated factors for both suicidal ideation and attempt. This result is concordant with other studies conducted in other Ethiopia, Japan, Pakistan, and Mozambique [43–47]. The possible reason for the association could be that being neglected by neighbors, friends, families, and others significantly lead to a feeling of hopelessness and worthlessness which finally leads to suicide [44, 45]. Getting support from peers and finding close friends highlight the role of social support in the mitigate the adverse effect of challenges of mental illness and other psychosocial developmental among youth [47]. The other reason might be because youth are mostly considered their friends, family member, and others significant as a source of emotional, advisor, and financial source of support at the time of crisis finally which leads to suicidal behaviors [48]. The other reason for association might be because they confine their challenge to their close is very easy and safe which gives protects from suicidal behavior.

The presence of anxiety was one of the major factors associated only with suicidal ideation but not with suicidal attempts. This finding is consistent with other studies conducted on European youth [49], The possible reason for the association might be due to because fright and fear of being mad finally lead to emotional stress that causes suicidal behaviors [49, 50]. The other reason for the association of anxiety could be evidenced by excessively worrying about many situations and the effect of frightening the situation they are worried about. Being anxious for unspecific tasks leads to hopelessness and worthlessness which ends with serious suicidal ideations.

## Limitation of the study

This study is not without drawbacks, the weakness of this paper is it can’t tell the temporal relationship between the outcome and determinant variables and the nature of the cross-sectional study design.

## Conclusions and recommendations

The prevalence of suicidal and/or attempt with substance abuse was high therefore, immediate interventions were needed since it is a very fatal and serious mental illness. It is recommended to decrease the consumption of substances to decrease the suicidal behaviors among youth particularly among females’ special attention needs to give since there are factors that increase the burden on females by the integration of zonal administrative and health bureau. The other recommendation for the zone community is for the social interaction functioning tried to be increased to mitigate the burden of anxiety and finally the burden of suicidal behaviors.

## Data Availability

All the data generated is included in the manuscript.

## Abbreviations

AOR: Adjusted Odd Ratio
ASSIST: Alcohol, Smoking, and Substance Involvement Screening Test
CI: Confidence Intervals
DASS: Depression Anxiety Stress Scales
DSM: Diagnostic and Statistical Manual of Mental Disorders
MSPSS: Multidimensional Scale of Perceived Social Support
WHO: World Health Organization
